# Estimation of the journal distance of *Genomics & Informatics* from other bioinformatics-driven journals, 2003-2018

**DOI:** 10.5808/gi.21074

**Published:** 2021-12-31

**Authors:** Ji-Hye Oh, Hee-Jo Nam, Hyun-Seok Park

**Affiliations:** 1 Bioinformatics Laboratory, ELTEC College of Engineering, Ewha Womans University, Seoul 03760, Korea; 2 Center for Convergence Research of Advanced Technologies, Ewha Womans University, Seoul 03760, Korea

**Keywords:** content analysis, document clustering, neural network, word embedding

## Abstract

This study explored the trends of *Genomics & Informatics* during the period of 2003-2018 in comparison with 11 other scholarly journals: *BMC Bioinformatics, Algorithms for Molecular Biology: AMB, BMC Systems Biology, Journal of Computational Biology, Briefings in Bioinformatics, BMC Genomics, Nucleic Acids Research, American Journal of Human Genetics, Oncogenesis, Disease Markers*, and *Microarrays*. In total, 22,423 research articles were reviewed. Content analysis was the main method employed in the current research. The results were interpreted using descriptive analysis, a clustering analysis, word embedding, and deep learning techniques. Trends are discussed for the 12 journals, both individually and collectively. This is an extension of our previous study (PMCID: PMC6808643).

## Introduction

*Genomics & Informatics* is the official journal of the Korea Genome Organization. A prototype version of the full-text corpus of *Genomics & Informatics*, called GNI version 1.0, has been recently archived in the GitHub repository [1,2]. In our previous study (PMCID: PMC6808643) [3], we conducted a statistical analysis of the publications of *Genomics & Informatics* over the 16 years since its inception, with a particular focus on issues relating to article categories, word clouds, and the most-studied genes, drawing on recent reviews of the use of word frequencies in *Genomics & Informatics* articles.

This paper is an extension of the work originally presented in *Genomics & Informatics*, vol. 17(3) [3]. Rather than exploring the trends of *Genomics & Informatics* alone, we intended to compare *Genomics & Informatics* with other representative biomedical or bioinformatics journals by measuring distances among journals and to explore the current trends in the field of biomedical research during the period of 2003‒2018.

Not all articles in PubMed Central (PMC) are available for text mining and other reuse; however, articles in the PMC Open Access Subset are made available for download under a license that generally allows more liberal redistribution and reuse than traditional copyrighted works [4]. We collected 22,423 available articles from the Author Manuscript Dataset, encompassing all articles collected under a funder policy in PMC and made available in machine-readable formats for text mining, from journals including *BMC Bioinformatics, Algorithms for Molecular Biology: AMB, BMC Systems Biology, Journal of Computational Biology, Briefings in Bioinformatics, BMC Genomics, Nucleic Acids Research, American Journal of Human Genetics, Oncogenesis, Disease Markers*, and *Microarrays* [5-16].

Content analysis was the main method employed to analyze the texts. A clustering algorithm and a shallow neural network were also used to interpret the interrelationship between keywords indicated in these articles. The content of the articles in the selected journals was processed according to categories derived from earlier studies.

Trends are discussed for the 12 journals, both individually and collectively. The findings obtained in this study may be useful in the exploration of potential research areas and the identification of neglected areas in the scope of *Genomics & Informatics*. The results were interpreted using descriptive analysis (frequencies). The reporting of the results was organized into the following categories: basic descriptive statistics, frequency analysis of selected genes, document clustering, and journal distance measurement.

## Basic Descriptive Statistics

The articles in the 12 journals were initially uploaded onto PubAnnotation, a project of the Database Center for Life Science) [17,18]. PubAnnotation provides a convenient way to add, annotate, and edit PMC publications based on the PMCID [17,18]. We specified the PMCIDs and uploaded the text files of the 12 journals.

Once a prototype corpus of the 12 journals has been constructed, we obtained basic descriptive statistics [19], which are statistics that do not seek to test for significance. The most basic statistical measure is a frequency count: a simple tallying of the number of instances of something that occurs in a corpus.

The importance of a term in each document is calculated based on weight functions and the entire collection of the document. Every document comprises particular words; therefore, this table creates a high-dimensional and sparse feature set, which brings tremendous noise to the text clustering and makes it difficult to appropriately cluster documents.

After preprocessing, a table was constructed based on the terms in the documents, presenting the occurrence of terms in each document and calculating their frequencies, as shown in Table 1.

According to Table 1, the number of articles published in each of the 12 journals ranged from 52 to 7,791. The average number of words per article of *Genomics & Informatics* was 3,011, which is the smallest among this group of journals; this reflects the fact that *Genomics & Informatics* has published a higher proportion of application notes and opinions than other journals. Issues of validity and reliability occur when the sample size of the study is too small given other factors. The number of articles in *Journal of Computational Biology* and *Microarray* was relatively small, so these data were normalized when necessary for later analysis.

Based on the existing database and the topological operation method for *Genomics & Informatics* obtained in our previous study [3], seven symbolic keywords were initially screened.

**Fig. 1.**
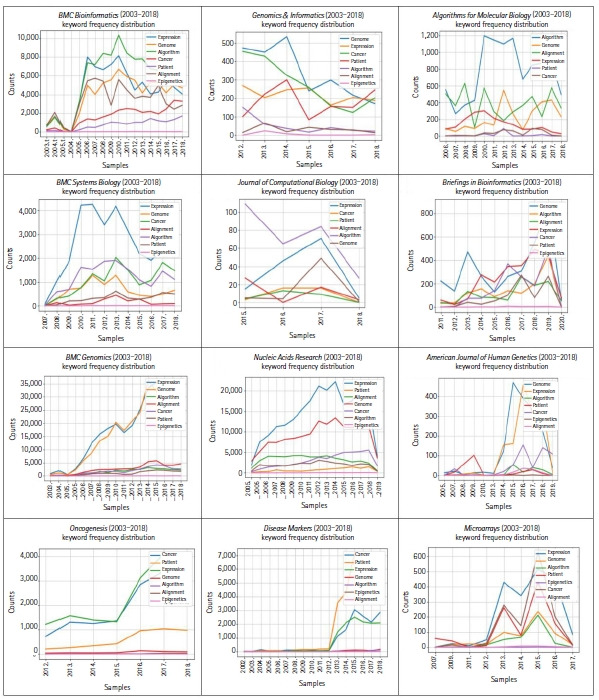
Conditional frequency distribution of exemplary keywords—algorithm, alignment, cancer, epigenetics, expression, genome, and patient—where the counts plotted are the number of times the word occurred in randomly chosen articles from 12 bioinformatics-related journals.

The plot in Fig. 1 is based on a conditional frequency distribution of these keywords—*algorithm, alignment, cancer, epigenetics, expression, genome*, and *patient*—where the counts plotted are the number of times the word occurred in each of these 12 journals. The keywords *algorithm* and *alignment* frequently appeared in *Algorithms for Molecular Biology*, which reflects the fact that the journal scope is bound to algorithms. The keyword *patient* appeared far more often in *Oncogenesis* and *Disease Markers*, which reflects the fact that these journals have a scope more oriented towards clinical pathology.

The frequency of gene names is another excellent measure of trends in the academic papers published in these journals. In our previous study, we compiled a list of the most-studied genes in publications listed in *Genomics & Informatics* from 2003 to 2018 [3]. The top 10 genes studied in *Genomics & Informatics* were: *EGFR*, *BRCA1*, *TP53*, *PIK3CA*, *BRCA2*, *PTEN*, *GAPDH*, *TNF*, *FTO*, and *APC*.

**Fig. 2.**
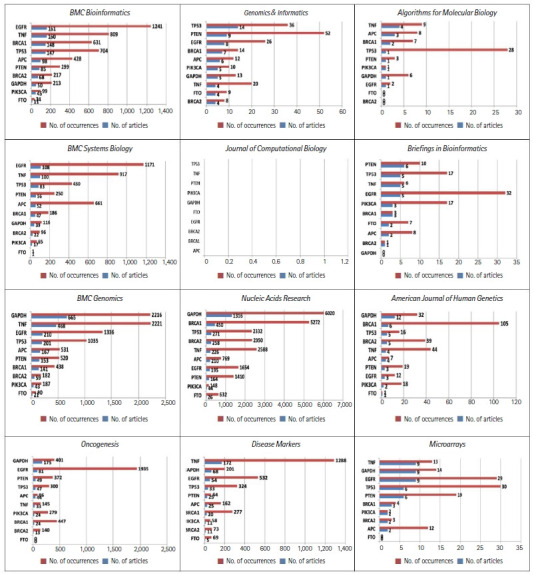
The frequency distributions of the top 10 genes in *Genomics & Informatics* in each of the 12 journals.

Fig. 2 shows the temporal dynamics of these top 10 genes over the years. Fig. 2 shows drastic differences in the frequency distribution of these genes in each of these 12 journals. For example, *EGFR* appeared 1,103 times in 99 different publications of *BMC Bioinformatics*. Considering that many genes have only appeared once in the journal, these remarkable frequency differences may reflect differences in the scope of the journals. In line with the same reasoning that the frequency distribution of the keyword patient indicates the scope of journals, as displayed in Fig. 1, the high frequency of genes related to human diseases also indicates journals’ scope. Noticeably, *PK3CA* appeared mostly in the *Journal of Computational Biology*. The *TNF* and *EGFR* genes frequently appeared in *BMC Systems Biology, Briefings in Bioinformatics, Disease Markers*, and *Oncogenesis*.

Almost all the most-studied genes are highly related to cancer, with the exception of *GAPDH*, a housekeeping gene. *GAPDH* appeared more often in *BMC Genomics, Nucleic Acid Research*, and *American Journal of Human Genetics*. In the more computation-oriented journals, such as *Journal of Computational Biology*, and *Algorithms for Molecular Biology*, the frequency of gene names was relatively rare.

## Document Clustering Based on Word Embedding Techniques

Another important measure of the scope of *Genomics & Informatics* in comparison to other journals is to classify the documents in an appropriate category and to compare the keywords to represent various cluster groups. Clustering is a useful technique that organizes a large quantity of unordered text documents into a small number of meaningful and coherent clusters, thereby providing a basis for an intuitive and informative evaluation of the characteristics of a journal. Our experiments utilized the standard K-means algorithm [20-22].

**Fig. 3.**
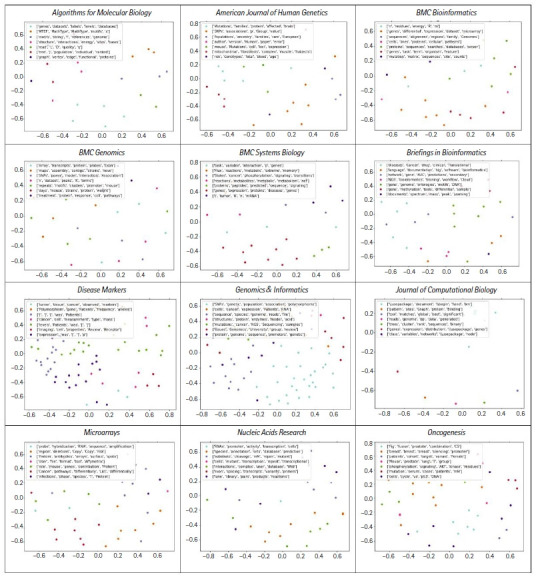
K-means clustering: comparison of seven clusters in 12 journals. Each dot represents a paper.

Fig. 3 shows K-means clustering, with clusters of articles published during the period from 2003 to 2018, displaying the data in a two-dimensional space. The well-known elbow method was used to identify the optimal number of clusters. The number of clusters was optimized only for *Genomics & Informatics*. Some drastic differences in the main topics for each journal were observed, as in Fig. 3. For example, the main topic of one of the clusters in *Algorithms for Molecular Biology* seems to be closely related to articles with a large proportion of programming code in the manuscript. However, the process of deciding main topics with clustering requires some human judgment and manual curation. This is one of the drawbacks of clustering, and it is beyond the scope of this paper.

## Journal Distance Estimation Based on a Shallow Neural Network

We estimated journal distances based on a shallow neural network [23]. To do this, we initially extracted 250,000 words from each of the 12 journals to measure distances among journals. Accurately representing the distance among documents has far-reaching applications. The most popular document representation methods have often relied on word embedding techniques such as the bag-of-words. In word2vec [24], one trains the model to find word vectors and then runs similarity queries between words. However, the bag-of-words approach can be problematic when the number of documents being represented is enormous, causing data sparseness problems.

To overcome this limitation of the bag-of-words approach, the doc2vec model [25], an extension of the word2vec method [24], has been made available. In doc2vec, one tags the text and obtains tag vectors. This method utilizes contextual information of each word and document to embed document vectors with manageable dimensionality into a continuous vector space.

**Fig. 4.**
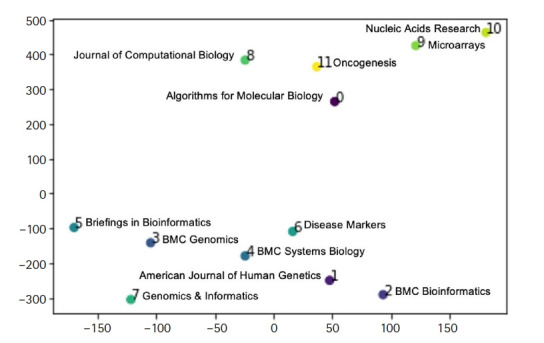
Journal distance estimation of the 12 journals.

Fig. 4 shows our estimation of journal distance of the 12 journals, using doc2vec and represented by t-distributed stochastic neighbor embedding [26]. The visual clusters can be influenced by the chosen parameterization. Fig. 4 shows that *Genomics & Informatics* is more closely related to computational genomics journals such as BMC Genomics than it is to pure bioinformatics journals such as *Journal of Computational Biology, Algorithms for Molecular Biology*, and *BMC Bioinformatics*.

## Summary

In this paper, the percentage frequencies of statistical procedures were compared between journals, providing original research findings based on a systematic collection and statistical analysis of research articles. The main findings of the study were the presence of considerably different profiles in terms of the statistical content among bioinformatics journals and the relationship of the scope of *Genomics & Informatics* with other journals. Although the scope of *Genomics & Informatics* covers a wide range of topics including gene discovery, comparative genome analyses, molecular and human evolution, informatics, genome structure and function, technological innovations and applications, statistical and mathematical methods, cutting edge genetic and physical mapping, and DNA sequencing, our analysis shows that *Genomics & Informatics* is more closely related to computational genomics journals than to pure bioinformatics journals. The findings obtained in this study may be useful in the identification of the journal scope and neglected areas of *Genomics & Informatics*.

## Figures and Tables

**Table 1.** Number of journal articles and words of the 12 journals 2003-2018

Journal list	Total No. of words	No. of articles retrieved	No. of words per article			
*Genomics & Informatics*	740,732	246	3,011.1			
*BMC Bioinformatics*	38,502,052	4,017	9,584.8			
*Algorithms for Molecular Biology: AMB*	6,112,875	167	36,604.0			
*BMC Systems Biology*	6,533,519	596	10,962.3			
*Journal of Computational Biology*	514,040	52	9,885.4			
*Briefings in Bioinformatics*	3,358,953	367	9,152.5			
*BMC Genomics*	67,798,532	6,835	9,919.3			
*Nucleic Acids Research*	66,442,455	7,791	8,528.1			
*American Journal of Human Genetics*	1,867,666	211	8,851.5			
*Oncogenesis*	4,600,049	579	7,944.8			
*Disease Markers*	6,522,158	1,483	4,397.9			
*Microarrays*	466,981	79	5,911.2			
